# Early Breast Cancers During Pregnancy Treated With Breast-Conserving Surgery in the First Trimester of Gestation: A Feasibility Study

**DOI:** 10.3389/fonc.2021.723693

**Published:** 2021-08-24

**Authors:** Concetta Blundo, Massimo Giroda, Nicola Fusco, Elham Sajjadi, Konstantinos Venetis, M. Cristina Leonardi, Elisa Vicini, Luca Despini, Claudia F. Rossi, Letterio Runza, Maria S. Sfondrini, Roberto Piciotti, Eugenia Di Loreto, Giovanna Scarfone, Elena Guerini-Rocco, Giuseppe Viale, Paolo Veronesi, Barbara Buonomo, Fedro A. Peccatori, Viviana E. Galimberti

**Affiliations:** ^1^Breast Surgery Unit, Fondazione IRCCS Ca’ Granda – Ospedale Maggiore Policlinico, Milan, Italy; ^2^Division of Pathology, IEO, European Institute of Oncology IRCCS, Milan, Italy; ^3^Department of Oncology and Hemato-Oncology, University of Milan, Milan, Italy; ^4^Division of Radiotherapy, IEO, European Institute of Oncology IRCCS, Milan, Italy; ^5^Division of Breast Surgery, IEO, European Institute of Oncology IRCCS, Milan, Italy; ^6^Division of Pathology, Fondazione IRCCS Ca’ Granda – Ospedale Maggiore Policlinico, Milan, Italy; ^7^Breast Imaging Unit, Fondazione IRCCS Ca’ Granda - Ospedale Maggiore Policlinico, Milan, Italy; ^8^Gynecology Unit, Fondazione IRCCS Ca’ Granda - Ospedale Maggiore Policlinico, Milan, Italy; ^9^Fertility and Procreation Unit, Division of Gynecologic Oncology, IEO European Institute of Oncology IRCCS, Milan, Italy

**Keywords:** breast cancer during pregnancy, pregnancy-associated breast cancer, early-onset breast cancer, early stage, breast-conserving surgery

## Abstract

Breast cancer is the most common malignancy occurring during gestation. In early-stage breast cancer during pregnancy (PrBC), breast-conserving surgery (BCS) with delayed RT is a rational alternative to mastectomy, for long considered the standard-of-care. Regrettably, no specific guidelines on the surgical management of these patients are available. In this study, we investigated the feasibility and safety of BCS during the first trimester of pregnancy in women with early-stage PrBC. All patients with a diagnosis of PrBC during the first trimester of pregnancy jointly managed in two PrBC-specialized Centers were included in this study. All patients underwent BCS followed by adjuvant radiotherapy to the ipsilateral breast after delivery. Histopathological features and biomarkers were first profiled on pre-surgical biopsies. The primary outcome was the isolated local recurrence (ILR). Among 168 PrBC patients, 67 (39.9%) were diagnosed during the first trimester of gestation. Of these, 30 patients (age range, 23-43 years; median=36 years; gestational age, 2-12 weeks; median=7 weeks; median follow-up time=6.5 years) met the inclusion criteria. The patients that were subjected to radical surgery (n=14) served as controls. None of the patients experienced perioperative surgical complications. No ILR were observed within three months (n=30), 1 year (n=27), and 5 years (n=18) after surgery. Among the study group, 4 (12.3%) patients experienced ILR or new carcinomas after 6-13 years, the same number (n=4) had metastatic dissemination after 3-7 years. These patients are still alive and disease-free after 14-17 years of follow-up. The rate of recurrences and metastasis in the controls were not significantly different. The findings provide evidence that BCS in the first trimester PrBC is feasible and reasonably safe for both the mother and the baby.

## Introduction

Breast cancer is the most common malignancy occurring in the course of gestation, with approximately 1,400 new diagnoses every year in Europe (1, 2). In the last decade, it has been observed a steady increase of breast cancer during pregnancy (PrBC) incidence (3–6). Delayed diagnosis is responsible for the worse outcome of PrBC compared to pregnancy-unrelated breast cancer, but stage-normalized survival is not different from that of age-matched non-pregnant controls (7–12). According to most guidelines, PrBC should be managed with the same protocols as breast cancer occurring in young non-pregnant women (1, 2, 13–18). However, the condition of pregnancy adds a layer of complexity to the treatment of PrBC because the benefit for the mother must not harm the fetus (2, 13, 19–22). For example, chemotherapy (CT) is contraindicated during the first trimester of gestation, while it can be safely administered in the second and third trimesters (23). Radiotherapy (RT) should be postponed until the postpartum period because of the risks associated with fetal radiation exposure (24, 25). Surgery is feasible and relatively safe at any stage of gestation, even if it might slightly increase the risk of pregnancy loss in the first trimester (1.0-2.0%) and might lead to premature birth in 1.5-2.0% of cases when performed in the second/third trimester (17, 26–28). Moreover, mastectomy is often proposed during the first trimester of pregnancy, regardless of the tumor stage, to reduce the risks of a delayed RT (14, 25, 26, 29–31). On the other hand, mastectomy is related to higher rates of post-surgical complications, psychological frailty, impairment of health-related quality of life, and increased sanitary costs (19, 30, 32–34). Regrettably, only a handful of studies on the specific outcome of PrBC patients treated with breast-conserving surgery (BCS) are currently available. Thus, the choice of the optimal surgical approach during the first trimester of pregnancy remains a matter of controversy.

In this study, we sought to provide evidence of the clinical feasibility and safety of BCS during the first trimester of pregnancy in women with early-stage PrBC.

## Materials and Methods

### Study Design

All patients with a diagnosis of PrBC during the first trimester of pregnancy at the European Institute of Oncology IRCCS and Fondazione IRCCS Ca’ Granda – Ospedale Maggiore Policlinico in Milan were included in this study. The first trimester was defined as 12 weeks and 6 days after the first day of the last menstruation. As for internal protocols, surgery followed the same conservative-oriented schemes applied for nonpregnant patients. Only women with early-stage PrBC treated with BCS during pregnancy followed by planned RT to the whole breast after delivery were included. The patients that were subjected to radical surgery (n=14) served as controls. Exclusion criteria for BCS were i) locally advanced disease; ii) history of breast cancer; iii) hereditary breast cancer; iv) multicentric disease; v) diffuse malignant microcalcifications on mammography; vi) inflammatory breast cancer; and vii) connective tissue disease. All cases underwent central pathological review at the Pathology Department of the European Institute of Oncology and were re-classified and re-graded following the latest World Health Organization criteria (35) and the Nottingham grading system (36). The staging was performed according to the American Joint Committee on Cancer (AJCC) Cancer Staging Manual (37). This study was approved by the local Institutional Review Board under protocol number #620_2018bis and was fully compliant with The Code of Ethics of the World Medical Association. Women were informed about the possible alternatives, including risks and benefits, and signed written informed consent.

### Characteristics of the Study Population

**Figure 1:**
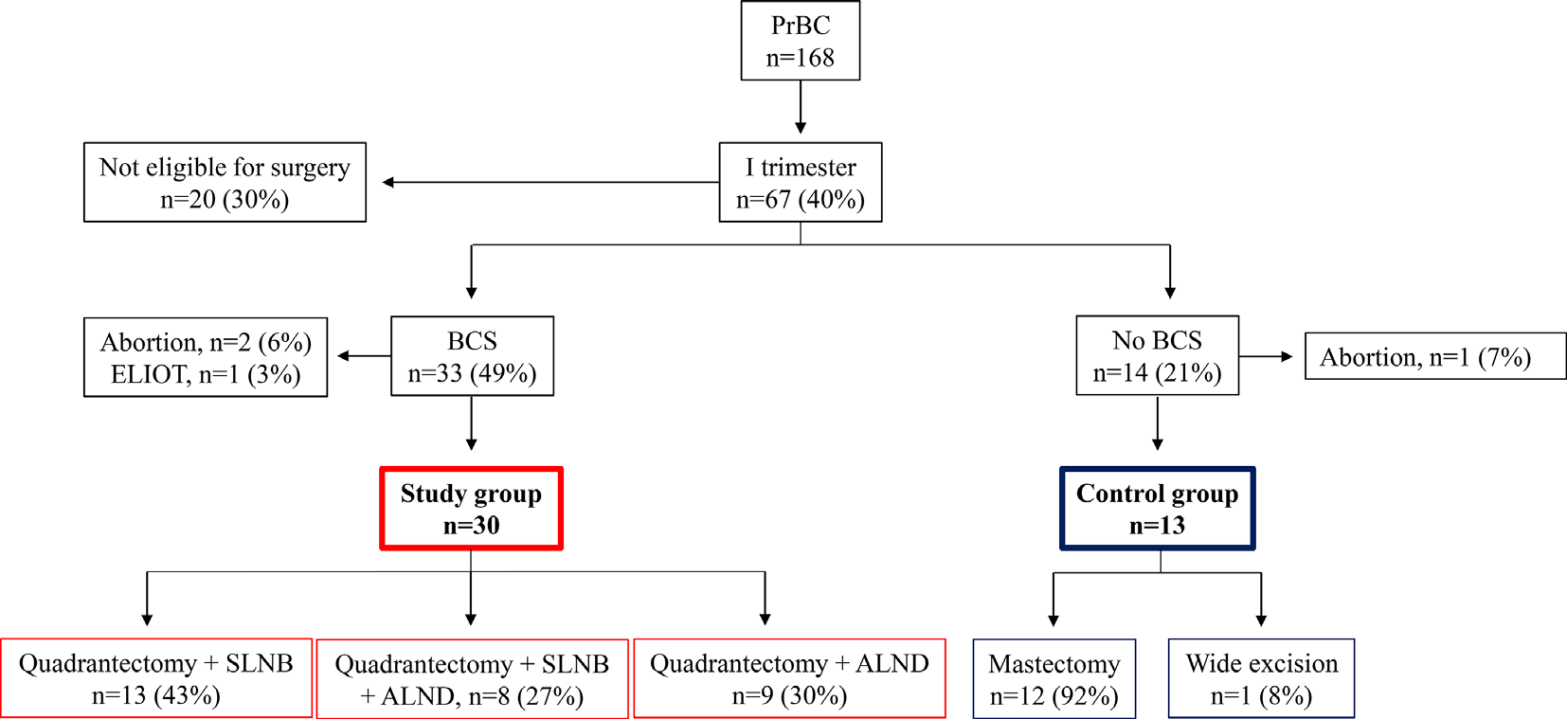
Study flowchart. PrBC, breast cancer during pregnancy. BCS, breast-conserving surgery; ELIOT, electron intraoperative radiotherapy; SLNB, sentinel lymph node biopsy; ALND, axillary lymph node dissection.

Taken together, 67 out of 168 (39.9%) PrBC patients treated from 2000 to 2020 had a diagnosis during the first trimester of gestation but 20 (29.9%) of them were not eligible for surgery due to locally advanced tumors. Of the remaining patients, 14 (20.9%) women were treated with mastectomy, including 2 that were subjected to a wide excision following a previous mastectomy. Apart from 1 (7.1%) patient who terminated her pregnancy, these no-BCS PrBC patients (n=13) represented our controls. Among the 33 PrBC treated with BCS during the first trimester, 3 were subsequently excluded; 2 because decided to terminate the pregnancy and received RT, and 1 because was enrolled in another study investigating the efficacy of electron intraoperative radiotherapy (ELIOT) (38). Thus, the study group was composed of 30 patients (age range, 23-43 years; median=36 years; gestational age at diagnosis, 2-12 weeks; median=7 weeks; median follow-up time=6.5 years). The study flow chart is portrayed in **Figure 1**, while the clinicopathological features of the patients included are shown in **Table 1**.

**Table 1:** Clinicopathologic features of the patients included in this study.

	Patients (n=30)
Age at diagnosis (Median ± SD)	36 ± 4.8
pT, n (%)	
1	21 (70.0)
2	6 (20.0)
Multifocal	3 (10.0)
pN, n (%)	
0	16 (53.3)
1	9 (30.0)
2	2 (6.7)
3	3 (10.0)
Histological subtype, n (%)	
NST (ductal)	25 (83.3)
Special types	5 (16.7)
Grade, n (%)	
1	4 (13.3)
2	9 (30.0)
3	17 (56.7)
LVI, n (%)	
Yes	13 (43.3)
No	17 (56.6)
Subgroup, n (%)	
ER+, HER2-	17 (56.6)
HER2+	3 (10.0)
ER-, HER2-	10 (33.3)
Ki67 status, n (%)	
High	20 (66.6)
Low	10 (33.3)

All cases were re-classified, re-graded, and re-assessed for hormone receptor, Ki67, and HER2 status according to the latest guidelines. SD, standard deviation; ER, estrogen receptor; HER2, human epidermal growth factor receptor 2; NST, no special type; LVI, lymphovascular invasion.

### Treatment, Follow-Up, and Outcome Evaluation

Before surgery, all participants were investigated with bilateral breast ultrasounds with the axillary examination. Bilateral mammography with a mediolateral-oblique view, +/- cranial-caudal investigation, was performed with fetal shielding. Standard pre-admission testing (e.g. electrocardiogram, blood pressure, laboratory blood work, chest X-ray with shielding, abdomen ultrasound) was performed 2-4 weeks before surgery. Genetic counseling and a thorough obstetrical and neonatological consultation were performed in all patients. After delivery, all patients received conventional 46-60 Gy RT to the ipsilateral breast. Owing to the potential teratogenic effects and increased miscarriage risk, patients were not given CT until the 12th week of pregnancy, when necessary. Both adjuvant endocrine therapy (ET) and target therapies (i.e. Tamoxifen, Aromatase inhibitors, gonadotropin-releasing hormone (GnRH) analogs, trastuzumab, and pertuzumab) were postponed after delivery, if appropriate. The primary outcome was the rate of isolated local recurrences (ILR) after delayed local irradiation of the breast.

## Results

### Delivery and Clinical Outcome

Thirteen (43.3%) BCS patients were concurrently subjected to sentinel lymph node biopsy (SLNB). Of these, 8 (26.7%) were positive and subsequent axillary lymph node dissection (ALND) was performed, while 9 (30.0%) women had clinically positive axilla and were subjected to ALND right away. Altogether, 21 (70.0%) PrBC were treated with adjuvant CT during gestation (including taxanes in 7 (33.3%) cases). The first RT dose was administered after 202-288 days from the primary surgery (median, 260 days) and after 23-101 days from the childbirth (median, 45 days). A total of 15 (50.0%) women had vaginal delivery; the median gestational age at the delivery was 36 weeks (range, 29-40 weeks). There were not reported fetal deaths nor congenital abnormalities of the newborns in both groups. The 5-year overall survival rate for all patients was 97% (n=29/30), as one patient died of metastatic disease after 33 months from the initial diagnosis.

### Isolated Local Recurrence-Free Survival

**Figure 2:**
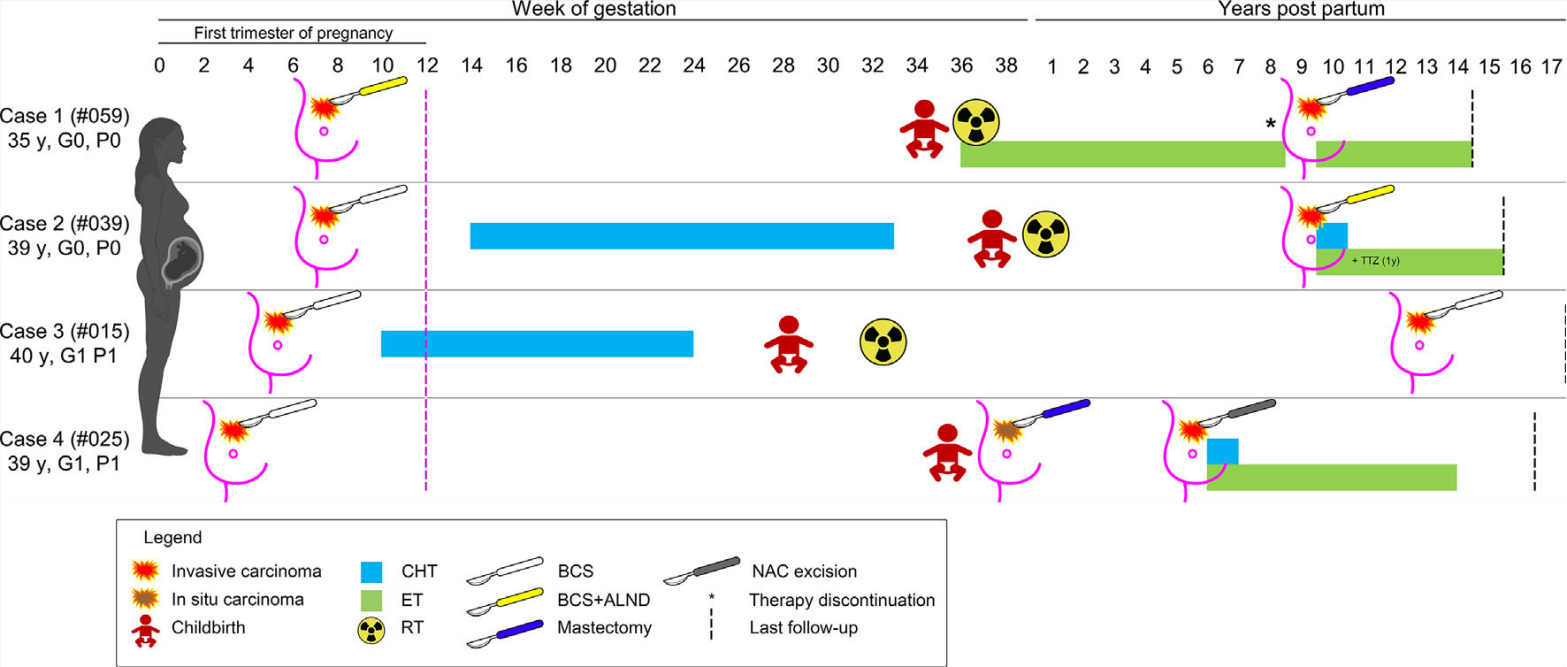
Schematic representation of the clinical history of the four patients with a diagnosis of breast cancer during pregnancy that experienced a secondary event after breast-conserving surgery. The timeline depicts the weeks of gestation and the years after childbirth, as reported on the top; patients are shown as rows, according to their ID and obstetric history on the left; the type of therapy is coded based on the legend on the bottom. CHT, chemotherapy; ET, endocrine therapy; RT, radiation therapy; TTZ, trastuzumab.

None of the patients from both groups suffered perioperative surgical complications. No ILR were observed within three months (n=30), 1 year (n=27), and 5 years (n=18) after BCS, while 4 (30.8%) controls had relapses after 3-7 years. Of note, four patients treated with BCS had ILR or new carcinomas after 6-13 years; these patients are still living and disease-free with a median follow-up time of 54 months (range 36-180 months). Their clinical history is detailed below and summarized in **Figure 2**.

#### Case 1

This patient (#PRBC059) was a 35-year-old Caucasian woman with no family and/or personal history of breast cancer. During the 7^th^ week of gestation, she was diagnosed with a malignant tumor of the left breast and was subsequently subjected to BCS, SLNB (positive), and ALND. Histopathological analyses revealed the presence of a bifocal pure mucinous carcinoma (paucicellular, i.e. type A) with hormone receptors (HR)+/HER2-/Ki67 16% phenotype, with low Ki67 index. BRCA testing revealed a wild-type gene status. The two nodules measured 0.5 cm and 1.5 cm in the greatest dimensions, and the final staging was pT1c(m) pN1(mi,1/34). The surgical margins were free from tumor cells. During the pregnancy period, the patient did not receive adjuvant therapy and gave birth at 34 + 5 weeks. Adjuvant RT and ET were commenced after delivery. Eight years later, the woman received two cycles of ovarian stimulation for a total period of 6 months, resulting in an ectopic pregnancy. New breast cancer in the ipsilateral breast was diagnosed 1 month after this event (ILR-free survival=103 months, 8.6 years). Therefore, the patient underwent a nipple-sparing mastectomy of the left breast and a risk-reducing mastectomy of the right breast with plastic reconstruction. The resected tumor was HR+/HER2- poorly differentiated (G3) invasive ductal carcinoma (IDC) measuring 0.7 cm in greatest dimensions (rpT1b) and no mucinous differentiation was observed. To date, the patient is alive, free of disease, and receiving ET.

#### Case 2

This patient (#PBC039) was a 39-year-old Caucasian woman with no family history of breast cancer. During the 7^th^ week of gestation, she was subjected to quadrantectomy and SLNB for a malignant tumor of the left breast. Histopathological analyses revealed the presence of a poorly differentiated (G3) HR+/HER2-/Ki67 35% IDC (pT1c pN0(sn)). The resection margins were negative. Starting from the 14^th^ week of gestation, CT was performed up to 33 weeks. Adjuvant RT followed the delivery at 38 weeks, but the patient refused the proposed ET. After 117 months, a new tumor was discovered in the ipsilateral breast. Compared with the former neoplasm, this tumor was HER2+. After BCS, combined CT, trastuzumab, and ET were administered. At present, the patient is alive 6 years after delivery, disease-free, and receiving ET.

#### Case 3

This patient (#PBC015) was a 40-year-old Caucasian woman, with no family history of breast cancer, who discovered a lump in her left breast. A core biopsy showed invasive breast cancer. During the pre-operative visit, a pregnancy test resulted in positive. Ultrasounds confirmed a gestational sac corresponding to 5 weeks of amenorrhea. Subsequently, she was subjected to left quadrantectomy and ALND and histology confirmed an IDC G3+ with an extensive intraductal component, free margins, negative lymph nodes, with a maximum diameter of 2.5 cm. The tumor had a triple-negative phenotype and the Ki67 index was 35%. During pregnancy, the patient received CT up to the 24th week and had a premature delivery at the 29^th^ gestational week. Subsequently, adjuvant RT was performed one month after delivery. No other medical therapies were performed. After 150 months, the patient developed a new ipsilateral tumor and was subjected to nipple-sparing mastectomy and contralateral reduction mammoplasty. This new tumor was a moderately differentiated triple-negative IDC. The patient has not undergone any type of adjuvant systemic therapy and is presently alive and disease-free after 17 years of follow-up.

#### Case 4

The patient (#PBC025) was a 39-year-old Caucasian woman with a family history of breast cancer. At the gestational age of 5 weeks, she was treated with BCS and SLNB for an IDC G1 with apocrine features measuring 3 mm in greatest dimensions, with associated ductal carcinoma *in situ* (DCIS) G2, and pleomorphic lobular carcinoma *in situ* (LCIS). The pathological staging was pT1a (is) pN0 (sn), and the tumor was a TNBC. The patient did not receive any adjuvant therapy during pregnancy. After delivery at the 36^th^ week, mammography and bilateral breast ultrasound revealed extensive microcalcifications. In the preoperative period, the patient had not performed bilateral mammography as per protocol, and 1 month after delivery, a histological examination confirmed the presence of DCIS G2. She underwent a nipple-areola complex (*NAC*) sparing mastectomy with radio-immuno-guided SLNB and plastic reconstruction. The pathology examination confirmed the presence of DCIS G2. After 64 months, she underwent removal of the nipple-areola complex due to the presence of CDI G2 with micropapillary aspects and extensive lymph-vascular invasion. Interestingly, the immunophenotype of the tumor was that of luminal breast cancer, with ER 40%, PgR40%, Ki67 15%, and HER2-.

## Discussion

PrBC represents an important health issue given its increasing incidence and the necessity of maintaining the balance between maternal and fetal well-being (39). The clinical management of this condition, especially in the first three months of gestation, is fairly challenging with limited therapeutic options (40). In this context, when the tumor is small, a conservative surgical approach could be employed as in the non-pregnant setting, also considering the possible complications after mastectomy, which could be particularly worrisome during pregnancy (41). Most patients received SLNB as part of the axillary investigation. The safety of ^99m^Technetium radiotracer for this procedure has been investigated in a dosimetry study, showing a very low dose associated with the lymphoscintigraphy procedure (in the range of 10-100 µGy) (42). Adjuvant RT to the whole breast is avoided throughout the pregnancy due to the risk of fetal radiation exposure. The fatal dose has been estimated to be up to 50 mGy in the first trimester and even higher in the late gestational age (43). This value exceeds the dose which is deemed not to be associated with measurable increased risk of fetal damage by the International Commission of Radiological Protection addressing the biological effects of prenatal irradiation (44). When adjuvant CT is indicated, the risk of delaying RT until the completion of systemic therapy is considered negligible (45). Therefore, it is generally applied the same approach as for non-pregnant, as the standard treatment of non-pregnant patients receiving CT is postponing RT (46). For those not receiving CT, the RT start should ideally be as close as possible to the surgery, shortly after childbirth. Hence, the local disease control is inversely proportional to the RT procrastination time, with a 1.14 relative risk of recurrence per month of delay (47). In the case of BRCA mutation, conservative treatment could be a bridge treatment of a more definitive intervention when the results of the test are available.

In our study, RT, ET, and anti-HER2 treatment were postponed after delivery (48). Local recurrences/second primary tumors were observed in 4 out of 30 patients treated with BCS. Given that patient #4 did not receive postoperative RT, but an after-delivery mastectomy for preoperative diagnostic underestimation during pregnancy, this case does not represent a post-BCS recurrence. On the other hand, cases #1-3 could be considered real relapses. Two of these tumors occurred in patients who received CT during pregnancy, in which the interval between the end of systemic therapies and the onset of RT was not influenced by the pregnancy. In a single patient (not eligible for systemic treatment in pregnancy), the RT was performed with a longer interval than the usual one of the non-pregnant patients. Survival was not affected by local relapse, underlining the efficacy of salvage treatment. Based on their IRC and subsequent salvage surgery, the 4 cases presented in detail here might represent a high-risk group of patients that requires particular attention in the choice of the surgical approach. Additional analyses encompassing not only clinical criteria but also molecular information would be required to precisely identify the early-stage PrBC at increased risk of relapse. Furthermore, due to the relatively small sample size of this feasibility study, and the subsequent impossibility of building a robust multivariable risk model, attention should be paid to the interpretation of our conclusions. It should be noted, however, that this is the first study providing previously unavailable data on BCS feasibility in this extremely rare condition. A randomized controlled multi-institutional trial would be required to address this question after controlling all other factors of the management armamentarium that can have an impact on the short- and long-term outcome, including delayed CT,

Despite these limitations, our results suggest that BCS during the first trimester of pregnancy in early-stage PrBC can be considered reasonably safe, providing the identification of women with low-risk clinical and biological features.

## Data Availability Statement

The original contributions presented in the study are included in the article/supplementary material. Further inquiries can be directed to the corresponding author.

## Ethics Statement

The studies involving human participants were reviewed and approved by IRCCS Ca’ Granda Ospedale Maggiore Policlinico. The patients/participants provided their written informed consent to participate in this study.

## Author Contributions

Study design, CB, MG, ML, and VG. Database curation, CB, NF, ES, KV, BB, and FP. First draft. CB, and MG. Initial review, NF, ES, KV, BB, and FP. Images, NF, ES, and KV. Bibliography, NF, ES, and KV. Supervision, NF, EGR, GV, PV, and VG. Critical review of the final draft, EV, LD, CR, LR, MS, RP, EL, and GS. All authors contributed to the article and approved the submitted version.

## Conflict of Interest

NF has received honoraria for consulting, advisory role, honoraria, travel, accommodation, and/or speaker bureau from Merck Sharp & Dohme (MSD), Boehringer Ingelheim, and Novartis. ER has received advisory fees from Novartis, Roche, and MSD Italia; honoraria from Thermo Fisher Scientific, AstraZeneca, Roche. GV has received honoraria for consulting, advisory role, speakers’ bureau, travel, accommodation, expenses, and/or research funding from MSD Oncology, Pfizer, Dako, Roche/Genetech, Astellas Pharma, Novartis, Bayer, Daiichi Sankyo, Menarini, Ventana Medical Systems Dako/Agilent Technologies, Cepheid, and Celgene. FP has received honoraria from Ipsen and Roche Diagnostics in the last 3 years.

The remaining authors declare that the research was conducted in the absence of any commercial or financial relationships that could be construed as a potential conflict of interest.

## Publisher’s Note

All claims expressed in this article are solely those of the authors and do not necessarily represent those of their affiliated organizations, or those of the publisher, the editors and the reviewers. Any product that may be evaluated in this article, or claim that may be made by its manufacturer, is not guaranteed or endorsed by the publisher.
